# New Exposure Location for Hantavirus Pulmonary Syndrome Case, California, USA, 2018

**DOI:** 10.3201/eid2510.190058

**Published:** 2019-10

**Authors:** Anne M. Kjemtrup, Sharon Messenger, Amy M. Meza, Tina Feiszli, Melissa Hardstone Yoshimizu, Kerry Padgett, Sunita Singh

**Affiliations:** California Department of Public Health, Sacramento, California, USA (A.M. Kjemtrup);; California Department of Public Health, Richmond, California, USA (S. Messenger, T. Feiszli, M. Hardstone Yoshimizu, K. Padgett);; County of Santa Cruz Public Health Department, Santa Cruz, California, USA (A.M. Meza);; Sutter Health Santa Cruz Center, Santa Cruz (S. Singh)

**Keywords:** hantavirus pulmonary syndrome, Peromyscus maniculatus, Sin Nombre virus, molecular epidemiology, viruses, vector-borne infections, California, United States

## Abstract

We describe a case of hantavirus pulmonary syndrome in a patient exposed to Sin Nombre virus in a coastal county in California, USA, that had no previous record of human cases. Environmental evaluation coupled with genotypic analysis of virus isolates from the case-patient and locally trapped rodents identified the likely exposure location.

Hantavirus pulmonary syndrome (HPS), which is caused by infection with Sin Nombre virus (SNV), was made nationally notifiable in the United States in 1995. Since then, 71 cases in California residents have been reported (range 0–8 cases/y) (*1*). Persons are usually exposed to SNV through inhalation of aerosolized excreta (e.g., saliva, urine, and feces) from infected rodents, typically deer mice (*Peromyscus maniculatus*) (*2*), although other wild mice, such as the cactus mouse (*P. eremicus*), have been implicated as reservoirs in California (*3*). SNV has been documented in deer mice throughout California (*4*), but exposure for most human cases has been in noncoastal, mountainous (>900 m elevation), rural areas of the state. 

Disease onset occurs after a 2–8-week incubation (*5*); onset for 70% of California cases has occurred during May–September. Laboratory confirmation includes serologic testing (e.g., ELISA, IgM, and IgG), reverse transcription PCR (RT-PCR) testing of serum or respiratory samples, or immunohistochemistry to identify virus antigen in tissue (*5*). Sequencing of viral RNA (most commonly glycoprotein or nucleoprotein open reading frames) is used to infer relationships of hantavirus strains from humans and rodents (*3*,*6*). We report a case of HPS in a patient exposed to SNV in a coastal county in California, USA, that had no previous record of human cases.

## The Study

The adult patient (age 35–40 years) sought care at a local urgent-care facility in April 2018 for a 2-day history of fever (38.8°C), chills, muscle aches, nausea, dizziness, shortness of breath, and cough. Initially diagnosed with a viral illness, the patient returned 2 days later with worsening symptoms, including nuchal rigidity and photophobia. The patient was referred to a local emergency department because of concerns about meningitis. 

At hospital admission, a computed tomography scan of the lungs showed bilateral perihilar air space consolidation, increased lung density (ground glass), and moderate bilateral pleural effusion. Clinical laboratory values demonstrated thrombocytopenia (≈46,000 cells/mL [reference 150,000–400,000 cells/mL]), elevated creatinine (1.5 mg/dL [reference 0.6–1.3 mg/dL]), elevated leukocytes (12,400 cells/mL [reference 3,900–11,700 cells/mL]), neutropenia (8.9% [reference 45%–70%]), and lymphopenia (17.3% [reference 18%–48%]). One day later, the patient was transferred to a hospital with a higher acute care level, intubated, and placed on extracorporeal membrane oxygenation. 

Serum collected 4 days after illness onset tested negative at the local public health laboratory for influenza A and B. At a commercial laboratory, serum test results were urine antigen–negative for leptospirosis and legionella, antibody-negative for coccidioidomycosis, and PCR-negative for *Yersinia enterocolitica* but were IgM- and IgG-positive for hantavirus. The California Department of Public Health Viral and Rickettsial Disease Laboratory tested an additional serum sample collected 7 days after illness onset, confirmed the positive serologic results, and detected SNV RNA by using a previously described RT-PCR (*7*). The patient remained on extracorporeal membrane oxygenation for 10 days, was extubated on day 18, and was released 20 days after illness onset.

The case-patient lives and works on a farm at ≈20 m elevation in Santa Cruz County, along the north coast of California. An interview with the family initially suggested a rural work exposure in San Mateo County, north of the farm residence, at ≈400 m elevation, where both the case-patient and a family member worked outdoors in a dusty, rodent-infested environment 18 days before illness onset. The family did not recall a substantial rodent exposure on the farm except for the case-patient cleaning a shed >1 week before illness onset.

The California Department of Public Health Vector-Borne Disease Section collaborated with county vector-control agencies to evaluate the case-patient’s place of residence, farm, and rural workplace for potential exposure to SNV. At the farm, rodent access, feces, and nesting material were present in multiple outbuildings and structures in and around the home. Of 105 Sherman traps set, 19 rodents were captured (18% trap success) from the farm, including 18 deer mice and 1 Western harvest mouse (*Reithrodontomys megalotis*). Rodents were anesthetized, bled through a retro-orbital blood collection technique, and humanely euthanized. Five (28%) of the deer mice and the harvest mouse were serologically positive for SNV, including 1 deer mouse from inside the shed that the case-patient cleaned and 1 from the basement of the house. Blood from 4 of the 5 deer mice and the harvest mouse were positive for SNV by RT-PCR.

The rural San Mateo County workplace location could not be investigated directly; however, trapping and habitat evaluation were conducted at public areas near the worksite. Rodents captured in 35 of 100 traps (35% trap success) included 15 parasitic mice (*P. californicus*) and 20 piñon mice (*P. truei*) but no deer mice. One piñon mouse tested serologically positive for SNV, but no viral RNA was detected by RT-PCR.

We conducted phylogenetic analysis to compare the case-patient’s isolate to other California hantavirus sequences, including those from the farm where the case-patient lived and worked. Because no PCR-positive rodents were collected near the rural worksite, archived sequences from SNV-positive deer mice collected in previous years (2014, 2016, and 2018) from 2 different sites in the same county as the rural worksite (San Mateo County) were included in our analysis. We found that the SNV glycoprotein sequence from the case-patient was genetically related most closely to the hantavirus sequences recovered from the case-patient’s farm (Figure). The sequences from the 2 sites in San Mateo County each form separate monophyletic clades that cluster together, despite collections over several years, and are distinct from all samples from Santa Cruz County. Thus, exposure most likely occurred at the farm where the case-patient lived and worked. Although the type of exposure of opening poorly ventilated outbuildings and performing activities that raise dust is typical for hantavirus exposure, the geographic location in this coastal California county has not been previously implicated in SNV exposure leading to HPS. Follow-up visits by county vectorborne disease officials provided information to the family on rodent exclusion and other prevention measures to reduce the risk for subsequent exposure to SNV.

**Figure:**
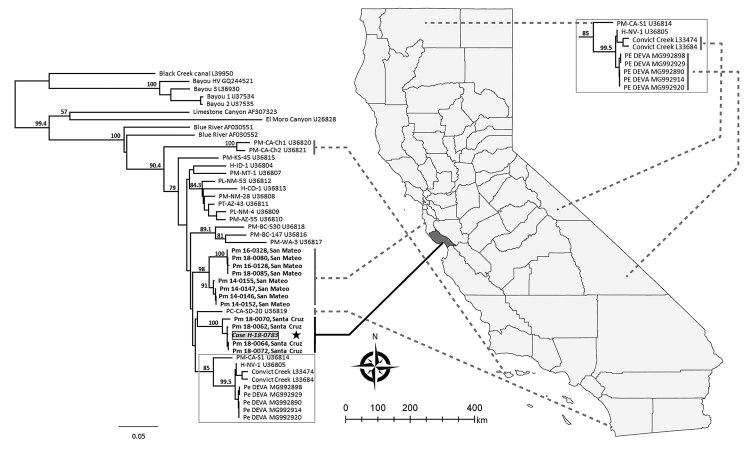
Phylogenetic tree of hantavirus Gn glycoprotein sequences from isolates collected in California, USA, and reference sequences. The hantavirus sequence from the case-patient described in this study (gray box) is shown in comparison to sequences from the case-patient farm in Santa Cruz County and archived samples from neighboring San Mateo County (bold). Dotted lines indicate general geographic origins of California sequences. Representative reference sequences of hantaviruses were downloaded from Genbank (accession numbers included in taxon labels). H indicates sequences from human cases; all other sequences are from small rodents. The tree was reconstructed by analysis of 848 bases of the glycoprotein precursor gene by using the neighbor-joining method and employing the Hasegawa-Kishino-Yano model to estimate genetic distances with Geneious 10.0 (https://www.geneious.com). We estimated support for relationships by using a nonparametric bootstrap analysis (1,000 replicates). Nodes with bootstrap percentages >50% are indicated. Similar tree topologies were generated from maximum-likelihood (RAxML) and Bayesian (Mr. Bayes) phylogenetic analyses (data not shown). Genbank accession numbers: Case H-18-0783, MK386451; Pm-18-0062, Santa Cruz, MK386452; Pm-18-0064, Santa Cruz, MK386453; Pm-18-0070, Santa Cruz, MK386454; Pm-18-0072, Santa Cruz, MK386455; Pm-14-0146, San Mateo, MK386456; Pm-14-0147, San Mateo, MK386457; Pm-14-0152, San Mateo, MK386458; Pm-14-0155, San Mateo, MK386459; Pm-16-0128, San Mateo, MK386460; Pm-16-0328, San Mateo, MK386461; Pm-18-0080, San Mateo, MK386462; and Pm-18-0085, San Mateo, MK386463. Scale bars represent the genetic distance (nucleotide substitutions per site).

## Conclusions

The prevalence of hantavirus in deer mice in the counties surrounding Santa Cruz ranged from 0% to 12% during 1975–2017 (*4*); however, human HPS cases have not been documented previously from this area. Typically, HPS cases are associated with higher elevations (*8*,*9*). The rural workplace was the first focus of this exposure investigation because it was at a higher elevation and the initial interview with the family suggested rodent exposure. Ultimately, however, the environmental investigation identified the most likely exposure location was the case-patient’s farm in Santa Cruz County. The high abundance of deer mice reported by the family, coupled with the presence of SNV in the mice found near the case-patient’s farm, likely contributed to elevated exposure risk. The environmental investigation of this case highlights the importance of evaluating all possible places of exposure to minimize future risk for illness and death from HPS. Molecular analysis of case-patient and rodent sequences was a valuable tool to identify the likely exposure locale.

The comprehensive epidemiologic investigation, including molecular sequencing, prompted public health messaging on hantavirus prevention to the public and medical community in a region where a hantavirus case had not previously been identified. Evaluation of the case-patient’s residence provided an opportunity for recommendations to decrease risk for ongoing exposure to the case-patient’s family. Findings from this environmental investigation might guide future public health interventions in California, including surveillance and public health messaging.
